# Novel Gram-Scale Production of Enantiopure *R*-Sulforaphane from Tuscan Black Kale Seeds

**DOI:** 10.3390/molecules19066975

**Published:** 2014-05-27

**Authors:** Gina Rosalinda De Nicola, Patrick Rollin, Emanuela Mazzon, Renato Iori

**Affiliations:** 1 Consiglio per la Ricerca e la sperimentazione in Agricoltura, Centro di Ricerca per le Colture Industriali (CRA-CIN), Via Di Corticella 133, Bologna 40128, Italy; 2 Institut de Chimie Organique et Analytique-UMR 7311, Université d’Orléans, Rue de Chartres, BP 6759, Orléans Cedex 2 45067, France; E-Mail: patrick.rollin@univ-orleans.fr; 3 IRCCS Centro Neurolesi “Bonino-Pulejo”, Via Provinciale Palermo, S.S.113, Contrada Casazza, Messina 98124, Italy; E-Mail: mazzon@irccsneurolesiboninopulejo.it or emazzon.irccs@gmail.com

**Keywords:** *R*-sulforaphane, Tuscan black kale, *Brassica oleracea*, glucoraphanin, glucosinolate, biphasic system

## Abstract

Dietary *R*-sulforaphane is a highly potent inducer of the Keap1/Nrf2/ARE pathway. Furthermore, sulforaphane is currently being used in clinical trials to assess its effects against different tumour processes. This study reports an efficient preparation of enantiopure *R*-sulforaphane based on the enzymatic hydrolysis of its natural precursor glucoraphanin. As an alternative to broccoli seeds, we have exploited Tuscan black kale seeds as a suitable source for gram-scale production of glucoraphanin. The defatted seed meal contained 5.1% (*w/w*) of glucoraphanin that was first isolated through an anion exchange chromatographic process, and then purified by gel filtration. The availability of glucoraphanin (purity ≈ 95%, weight basis) has allowed us to develop a novel simple hydrolytic process involving myrosinase (EC 3.2.1.147) in a biphasic system to directly produce *R*-sulforaphane. In a typical experiment, 1.09 g of enantiopure *R*-sulforaphane was obtained from 150 g of defatted Tuscan black kale seed meal.

## 1. Introduction

**Scheme 1:**
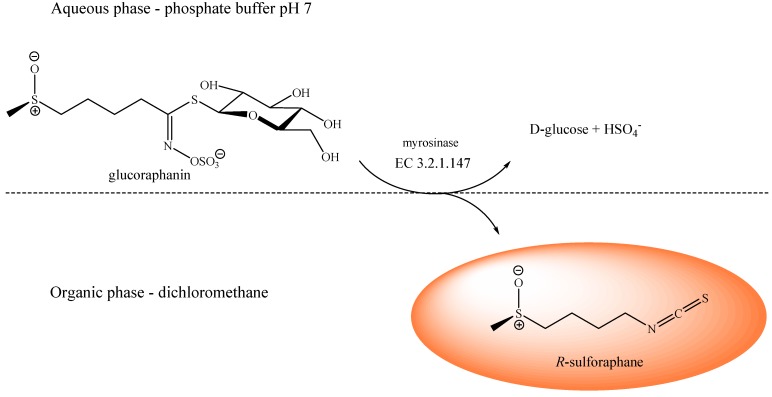
Production of enantiopure *R*-sulforaphane. Myrosinase(β-thioglucoside glucohydrolase; E.C. 3.2.1.147)catalyzed hydrolysis reaction of glucoraphanin (GRA), purified fromTuscan black kale defatted seed meal, in a biphasic system. Upper layer: phosphate buffer 0.3 M pH 7.0, lower layer: dichloromethane.

Since dietary sulforaphane (4*R*-1-isothiocyanato-4-(methylsulfinyl)butane; *R*-sulforaphane; CAS RN [142825-10-3]) was isolated from broccoli [1], several hundred papers have focused on this isothiocyanate (ITC), which is one of the most potent naturally occurring inducers of the Keap1/Nrf2/ARE pathway [2]. Furthermore, sulforaphane is currently being used in clinical trials to assess its effects against different tumour processes [3]. *R-*Sulforaphane is not produced as such by the plant, but rather released by the enzymatic action of myrosinase (β-thioglucoside glucohydrolase; E.C. 3.2.1.147) on the glucosinolate (GL) precursor glucoraphanin (GRA; 4-methylsulfinylbutyl GL) following cell damage. The configuration of the sulfoxide stereogenic center in the GRA side chain (Scheme 1) was recently ascertained by NMR to be *R_S_*, a configuration retained in the hydrolysis product sulforaphane [4]. It has to be clarified that the stereochemical absolute configuration of natural sulforaphane has been established unequivocally: the incorrect and confusing designation “l-sulforaphane” currently found in many articles [5] clumsily refers to the levorotatory nature of the compound.

Up to now, most studies on the biological activities of sulforaphane have been conducted using *R*,*S*-sulforaphane, the racemic form obtained by chemical synthesis, as early studies had shown that the chirality of the sulfoxide group in sulforaphane did not affect its potency as an enzyme inducer [1].

Racemic sulforaphane was first synthesized in Zürich by Schmid and Karrer [6]. A procedure to produce *R*,*S*-sulforaphane on the gram scale was developed later [7]. This was based on the myrosinase-catalyzed hydrolysis of hemi-synthetic GRA resulting from chemoselective oxidation of glucoerucin (GER; 4-methylsulfanylbutyl GL) extracted from rocket seed. More recently, a six-step synthetic method suitable for multi-gram scale production of racemic sulforaphane with a 64% overall yield was reported [8]. As humans are exposed exclusively to *R*-configurated sulforaphane through the diet, investigations of the effectiveness of this enantiomer to modulate carcinogen-metabolizing enzymes should be carried out in comparison with the *S*-enantiomer and with the racemic mixture. As a matter of fact, marked differences in the modulation of cytochrome P450 and of the phase II enzymes, by the two enantiomers of sulforaphane, have been recently reported. In precision-cut tissue slices of both liver and lung of rats, *R-*sulforaphane enhanced quinone reductase and glutathione *S*-transferase activities, whereas *S*-sulforaphane either was ineffective or provoked a much weaker response [9]. Future investigations in animal or clinical trials on the pharmacological properties of *R-*sulforaphane, as a promising natural compound in chemopreventive therapy, are suggested in recent reviews of existing studies [10,11]. There is therefore a need for making available large quantities of enantiopure *R*-sulforaphane. Several methods for the purification of natural *R-*sulforaphane are based on the separation of the compound from complex mixtures of ITCs present in water extracts of broccoli seed or seed meal after myrosinase hydrolysis. Those methods are based on low-pressure column chromatography [12], solid phase extraction coupled with preparative HPLC [13] and macroporous resin [14], but generally, the yield and/or purity of the obtained *R-*sulforaphane were limited. 

The present report discloses an efficient methodology for the production of enantiopure *R*-sulforaphane based on the enzymatic hydrolysis of its natural precursor GRA, which in turn has to be extracted and purified from an appropriate vegetable source. In a recent report, the GLs content of 32 cultivars of cabbage and 24 cultivars of kale was analysed [15]. The authors mentioned in particular five cultivars of kale from Italy, categorized as black kale (*Cavolo nero*), as excellent sources of GRA, hence as potential plant materials to produce sulforaphane. Accordingly, as an alternative to broccoli seeds, we have exploited the seeds of Tuscan black kale (*Brassica oleracea* (L.) ssp *acephala* (DC) var. *Sabellica* L.) as a suitably rich source for the production of GRA [16]. The gram-scale availability of purified GRA has thus allowed us to develop a novel hydrolytic process involving myrosinase in a biphasic system (Scheme 1) to produce enantiopure *R-*sulforaphane as a single product in 99% yield without the need of any further chromatographic steps. 

## 2. Results and Discussion

### 2.1. Purification of Glucoraphanin from Tuscan Black Kale Seeds

**Figure 1:**
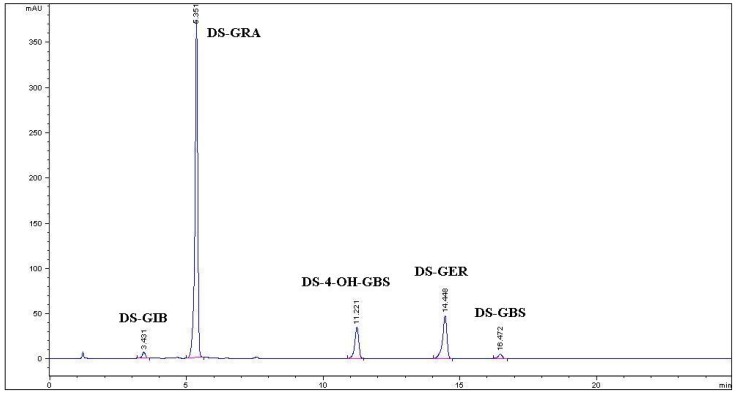
HPLC chromatogram of desulfo-glucosinolates (DS-GLs) isolated from an ethanolic extract of Tuscan black kale defatted seed meal. DS-GIB: desulfoglucoiberin; DS-GRA: desulfoglucoraphanin; DS-4-OH-GBS: desulfo-4-hydroxyglucobrassicin; DS-GER: desulfoglucoerucin; DS-GBS: desulfoglucobrassicin.

Individual and total GLs content in Tuscan black kale defatted seed meal are reported (Table 1), while Figure 1 shows a typical HPLC chromatogram of desulfo-GLs (DS-GLs) isolated from an ethanolic extract. Three aliphatic GLs bearing a thio-functionalized side chain accounted for 95% of the total content with predominant GRA, followed by GER and a minor amount of glucoiberin (GIB; 3-methylsulfinylpropyl GL). Two indolic GLs, namely 4-hydroxyglucobrassicin (4-OH-GBS; 4-hydroxy-3-indolylmethyl GL) and glucobrassicin (GBS; 3-indolylmethyl GL), were also present, accounting for the remaining 5% of total GLs. The starting defatted seed meal was extracted with boiling 70% ethanol with a GLs extraction yield of 78.4%. The GLs mixture isolated after anion exchange chromatography and precipitation with cold absolute ethanol consisted of GRA in a purity of 81.1% (*w/w*), the rest being GIB 1.4% (*w/w*), GER 6.7% (*w/w*), and most likely potassium sulfate and yellow substances which were all eliminated further on by gel filtration (Table 1). Enhancement of the purity level of GRA was achieved by the final gel filtration step. Seven runs of gel filtration afforded 3.10 g of purified GRA as a white amorphous solid after freeze-drying. The purity of GRA assessed by HPLC resulted to be 99% (area peak based) and 95% on weight basis. 

**Table 1:** Summary of data for the purification of glucoraphanin (GRA) and the production of *R*-sulforaphane starting from 150 g of Tuscan black kale (*Brassica oleracea* (L.) ssp *acephala* (DC) var. *Sabellica* L. cv. 0D74) defatted seed meal.

Purification Step	Amount	Aliphatic GLs			Indole-Type GLs		Total GLs
		GIB	GRA	GER	4-OH-GBS	GBS	
TBK-DSM	150 g	0.15 (±0.01) ^a^	7.66 (±0.62)	1.25(±0.17)	0.46 (±0.05)	0.01 (±0.00)	9.53 (±0.85)
Ethanolic extract	2.29 L	0.10 (±0.00)	6.20 (±0.02)	0.88(±0.00)	0.19 (±0.07)	0.10 (±0.00)	7.47 (±0.09)
GLs mix powder (from DEAE A-25)	7.19 g	0.10 (±0.00)	5.83 (±0.14)	0.48(±0.06)	−	−	6.41 (±0.20)
Purified GRA	3.10 g						
*R*-Sulforaphane	1.09 g						

^a^ Glucosinolate content (g) reported as the mean of eight determinations (*n* = 8). Numbers in parenthesis denote the standard deviation (±SD). Legend: TBK-DSM: Tuscan black kale defatted seed meal; GIB: glucoiberin; GRA: glucoraphanin; GER: glucoerucin; 4-OH-GBS: 4-hydroxy glucobrassicin; GBS: glucobrassicin.

### 2.2. Glucoraphanin Molar Extinction Coefficient Determination

**Figure 2:**
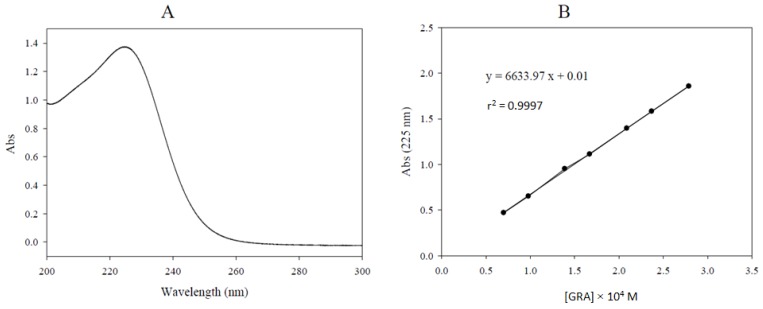
UV spectrum of purified glucoraphanin (GRA) (purity 95%) in water at a concentration value of 2.089 × 10^−4^ M. λ_max_: 225 nm (**A**). Absorbance *vs.* glucoraphanin (GRA) concentration plot used for the determination of the molar extinction coefficient (ε) in water at 225 nm. The ε calculated by linear regression as the slope of the plot resulted 6634 M^−1^ cm^−1^ (**B**).

The UV spectrum of GRA was registered between 200 and 320 nm (Figure 2A) in water solution, where a maximum absorption at 225 nm is exhibited. The ε (225 nm, water) measured value of GRA, potassium salt, was 6634 M^−1^ cm^−1^ (Figure 2B). This value can be of considerable importance to the analytical chemist in correcting the purity level as already pointed out [17]. Our value greatly differs from that already reported: in that previous work [18] a ε value of 6872 M^−1^ cm^−1^ was determined for GRA, being measured in water at 235 nm, which is not the λ_max_ for this GL.

### 2.3. Production of Enantiopure R-Sulforaphane

The myrosinase catalyzed hydrolysis of highly purified GRA (purity 95%, 3.10 g, 6.192 mmol) was performed in a phosphate buffer/dichloromethane biphasic system. Enantiopure *R-*sulforaphane (1.09 g, 6.130 mmol, 99% yield) was recovered from the dichloromethane phase as a light yellow oil and characterized as reported in the following sections. 

### 2.4. R-Sulforaphane Characterization

HPLC, t*_R_* = 5.8 min. 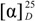 −76 (c 1.3, CHCl_3_); lit. data: −78.2 (c 0.6, CHCl_3_) [19]. GC-MS, t*_R_* = 18.7 min; EIMS 70 eV *m/z* (rel. int.): 72 (100), 160 (64), 55 (43), 39 (15), 45 (13), 64 (12), 63 (10), 41(10), 114 (8), 74 (6). The observed data are in agreement with literature values [20]. IR (cm^−1^): 3426 (O-H from H_2_O adsorbed), 2923, 2867 (C-H), 2179, 2100 (N=C=S), 1451, 1349 (C-H), 1260 (C-N), 1021 (S=O), 739 (C-H), 688 (C-S), in accordance with literature data [21]. ^1^H-NMR (400 MHz, CDCl_3_) δ (ppm): 3.59 (t, *J_vic_* = 6.0 Hz, 2H, CH_2_N), 2.80–2.66 (m, 2H, CH_2_S), 2.59 (s, 3H, CH_3_), 1.97–1.83 (m, 4H, CH_2_CH_2_). ^13^C-NMR (100 MHz, CDCl_3_) δ (ppm): 53.6 (CH_2_S), 44.8 (CH_2_N), 38.7 (CH_3_), 29.1 (C-2), 20.2 (C-3). NMR data were in accordance with literature data [19].

### 2.5. Discussion

Tuscan black kale seed has shown to be a valuable vegetable source of GRA, particularly suitable for purification purposes. In fact the defatted seed meal contained a high percentage (5.1% *w/w*) of GRA which was also the most abundant among a limited number of only five GLs, representing 80% of the total GL content. Those data fulfilled the starting conditions to make it a remarkable candidate amongst brassica vegetables for an efficient purification of GRA through a simple procedure. Although broccoli seeds have also been reported to be a good source of GRA, the investigated Tuscan black kale seeds showed a higher amount of GRA compared with different commercial broccoli seeds cultivars that were obtained locally. Moreover, GRA only constitutes approximately 50% of the GLs present in broccoli, which represents a major drawback for the purification of this GL [22]. In our study, we present a complete and detailed strategy to purify GRA via a two-step chromatographic process starting from Tuscan black kale defatted seed meal. The purification procedure involves the initial inactivation of myrosinase by use of boiling 70% ethanol. After extraction, GLs are isolated using an anion exchange column chromatography on DEAE Sephadex A-25, well adapted for optimum recovery of the loaded mixture of methionine-derived GLs, namely GIB, GRA and GER, by eluting with a 0.2 M aqueous solution of potassium sulfate. The DEAE Sephadex A-25 resin proved to be suited to our purpose, further allowing isolation of the GLs mixture as a fine powder with a good purity level of 81.1% after precipitation with cold absolute ethanol. Both indole-type GLs (4-OH-GBS and GBS) are retained by the resin and can therefore be eluted at higher salt concentration [23]. After the first chromatographic step, GRA is further purified by gel filtration on Sephadex G-10, which enables to discard the yellow contaminants present in the starting mixture, and to separate GIB and GER from GRA. The gram-scale availability of highly pure GRA allows one to design a strategy to easily produce amounts of the naturally occurring enantiopure *R*-sulforaphane. Although natural sulforaphane exists as a single enantiomer with a *R_S_* absolute configuration, most studies on its biological activities have been conducted by using the racemic form. As humans are exposed exclusively to *R*-sulforaphane through the diet, investigations of the effectiveness of this enantiomer to modulate carcinogen-metabolizing enzymes should be carried out in comparison with the *S*-enantiomer and with the racemic mixture. Recently, the potential of *R*-sulforaphane, *S*-sulforaphane and *R,S*-sulforaphane to modulate two major carcinogen-metabolizing enzymes, namely glucuronosyl transferase and epoxide hydrolase, was investigated in precision-cut rat liver slices. The results of this study have shown that the ability to enhance the activities of the two enzymes is dependent on the stereogenicity of the sulfoxide moiety. It was observed that the *R*-enantiomer increased both activities, whereas, in contrast, the *S*-enantiomer impaired them, the racemic mixture being inactive in this respect [24]. The naturally occurring *R*-enantiomer is a potential chemopreventive agent and it has proven furthermore to be a promising molecule for the prevention/treatment of neurological diseases in an animal model of multiple sclerosis and Parkinson’s disease [25,26]. It is therefore important to have in hand a simple and efficient method for the preparation of enantiopure *R*-sulforaphane. It is worth mentioning that the novel method disclosed herein conveniently allows gram-scale production of the target compound without requiring any purification steps. Sulforaphane is usually regarded as a labile compound and moreover, because of its poor solubility in water, several difficulties are met during *in vitro* and *in vivo* testing of sulforaphane to correctly assess its biological properties. To overcome these drawbacks, our strategy of producing large amounts of *R*-sulforaphane can be positively combined with already reported effective methods to enhance its stability such as the inclusion in cyclodextrins [21,27] or its formulation in polyethylene glycol successfully applied for topical applications [28].

## 3. Experimental

### 3.1. Chemicals

The resins diethylaminoethyl (DEAE) Sephadex A-25 and Sephadex G-10 were purchased from GE Healthcare (Freiburg, Germany). Acetonitrile, methanol and dichloromethane were HPLC grade from Sigma-Aldrich Chemie (Steinheim, Germany), whereas other chemicals were of analytical grade. Ultrapure water was obtained from a Milli-Q Gradient instrument (Millipore SAS, Molsheim, France) equipped with a Millipack filter 0.22 µm (Millipore, SAS).

The enzyme myrosinase (β-thioglucoside glucohydrolase, E.C. 3.2.1.147) was isolated from *Sinapis alba* L. seeds as described [29] with some modification. The specific activity of the stock solution used in the present study was 60 U mg^−1^ of soluble protein. The enzymatic activity was 12 U mL^−1^ and the solution was stored at 4 °C in sterile distilled water until use. One myrosinase unit was defined as the amount of enzyme able to hydrolyze 1 µmol sinigrin (SIN; 2-propenyl GL) per minute at pH 6.5 and 37 °C. 

### 3.2. Plant Source

Ripe seeds of Tuscan black kale (*Brassica oleracea* (L.) ssp *acephala* (DC) var. *Sabellica* L. cv. 0D74) were supplied in 2011 by Suba Seeds Company (Longiano, FC, Italy) and stored in a dry and dark place at room temperature. Seeds were identified by a lot number and guaranteed by the producer for the quality and the homogeneity of the product.

### 3.3. Glucoraphanin Extraction and Purification

GRA was isolated from Tuscan black kale (*Cavolo nero di Toscana*) ripe seeds, following a slight modification of the procedure developed at CRA-CIN (Bologna, Italy) and briefly reported in a previous article [16]. Seeds were first ground to a fine powder and defatted overnight in hexane. A sample of dried defatted seed meal (150 g) was treated with boiling 70% ethanol (700 mL) in order to quickly deactivate the endogenous myrosinase. GLs were extracted using an Ultraturrax homogenizer T50 at medium speed for 20 min. The resulting homogenate was centrifuged at 14,000 *g* for 30 min and the extraction repeated on the solid as before. The two extracts were pooled, and 0.5 M acetate buffer pH 4.2 (100 mL) was added and diluted with water (up to a final volume of 3 L). The treated extract was left overnight at 4 °C for protein precipitation. The isolation of GLs from the extract was carried out by one-step ion exchange chromatography. The extract was filtered and loaded on a glass column (Econo-Column 2.5 × 20 cm, Bio-Rad Laboratories, Milan, Italy) packed with DEAE Sephadex A-25 anion exchanger (90 mL) conditioned with 25 mM acetate buffer (pH 4.2). After washing with distilled water (2 L), the GLs were eluted with a 0.2 M aqueous solution of potassium sulfate (500 mL). The collected solution was concentrated to dryness using a rotary evaporator Laborota 4002 (Heidolph Instruments, Schwabach, Germany). The solid residue was then submitted to three subsequent extractions with boiling methanol (3 × 100 mL). The alcoholic extracts were then filtered and concentrated by rotary evaporation to about 10% of the initial volume. Afterwards, the solution was warmed, and slowly added dropwise under stirring to absolute ethanol (≥99.8%, 200 mL) previously cooled to −20 °C, leading to the precipitation of a white powder. After centrifugation, the solid was thoroughly dried under vacuum, then reduced to a fine powder and sealed under reduced pressure to prevent moisture uptake. The purity of GRA was further improved by gel filtration performed on a XK 26/100 column packed with Sephadex G-10 connected to an AKTA fast protein liquid chromatograph system (FPLC) (GE Healthcare, Milan, Italy). The isolated GLs powder was dissolved in ultrapure water (0.5 g mL^−1^), filtered through a 0.45 µm membrane filter (Gema Medical S.L., Barcelona, Spain), charged (2 mL) onto the column and eluted using a mobile phase of ultrapure water at a flow rate of 2.0 mL min^−1^ monitoring the absorbance at 254 nm. Individual fractions (6 mL) of seven runs were analyzed by HPLC and those containing pure GRA were pooled and freeze-dried. GRA (potassium salt; MW 475.59) was characterized by ^1^H- and ^13^C-NMR and the purity was assayed by HPLC analysis of its desulfo-derivative according to the EU official ISO 9167-1 method [30], as described in Subsection 3.5.

### 3.4. R-Sulforaphane Production

A sample of purified GRA (95% weight based, 3.10 g; 6.192 mmol) was dissolved in potassium phosphate buffer pH 7.0 (0.3 M, 120 mL) and mixed with dichloromethane (160 mL). After addition of myrosinase (1 mL, 12 U mL^−1^), the mixture was vigorously stirred at 37 °C overnight. After cooling at room temperature, the organic phase was decanted and the aqueous phase extracted with dichloromethane (3 × 10 mL). The organic layers were pooled, dried over anhydrous sodium sulfate_,_ and the solvent was then removed by rotary evaporation at room temperature. The oily residue was sealed under reduced pressure and stored at −28 °C.

### 3.5. Glucosinolate Profiling and Quantification by HPLC-PDA Analysis

GLs were extracted from duplicate samples (about 100 mg) of finely powdered defatted seed meal, as previously reported [31]. Each extract (1 mL) was loaded onto a mini-column filled with DEAE Sephadex A-25 anion exchanger (0.6 mL), conditioned with 25 mM acetate buffer pH 5.6. After washing with the same buffer (3 mL), purified sulfatase (100 μL, 0.26 U mL^−1^) was loaded onto the mini-column which was left overnight at room temperature. The desulfo-GLs (DS-GLs) were then eluted with ultrapure water (3 mL) and finally injected into an HPLC Agilent 1100 system equipped with a PDA detector and an Inertsil ODS-3 column (250 × 3.0 mm, 5 μm) thermostated at 30 °C. The chromatography and the quantification were achieved as reported [32]. DS-GLs were detected by absorbance monitoring at 229 nm. The amount of GL was quantified by using a calibration curve of pure DS-SIN solution (range from 0.14 to 1.4 mM) and the relative proportionality factors (RPFs) of each individual DS-GL [17]. All desulfation procedures were carried out in quadruplicate. 

### 3.6. Glucoraphanin Molar Extinction Coefficient Determination

The molar extinction coefficient (ε) of GRA was determined by using a computerized Varian Cary 300 Bio UV/Visible spectrophotometer (Varian, Palo Alto, CA, USA) equipped with 1 cm quartz cells. A stock water solution of a purified GRA sample (95% weight based, 10.46 mg mL^−1^) was prepared and 10, 14, 24, 20, 30, 34 and 40 µL aliquots were diluted into the quartz cell up to 3 mL with water to achieve seven concentration levels from 0.70 × 10^−4^ to 2.79 × 10^−4^ M. The absorbance of each final solution was measured at 225 nm in triplicate against a water blank at 25 °C. Absorbance mean values were plotted against the corresponding molar concentrations and the ε calculated by linear regression as the slope of the plot.

### 3.7. NMR Analysis of Glucoraphanin and Sulforaphane

^1^H- and ^13^C-NMR spectra were recorded on a 400 MHz Avance 2 spectrometer (Bruker Biospin SA, Wissembourg, France). GRA was dissolved in deuterated water, *δ* values being referenced to DOH at 4.80 ppm. *R*-sulforaphane was dissolved in deuterated chloroform, *δ* values being referenced to residual CHCl_3_ at 7.26 ppm. 

### 3.8. HPLC-PDA Analysis of Sulforaphane

Pure *R*-sulforaphane was dissolved in 10% aqueous acetonitrile and analyzed using an Agilent 1100 HPLC system (Agilent, Waldbronn, Germany) with an Inertsil ODS-3 column (250 × 3.0 mm, 5 μm particle size), thermostated at 30 °C, and equipped with a PDA detector. The chromatography was performed at a flow rate of 0.8 mL min^−1^ eluting with a gradient of H_2_O (A) and acetonitrile (B) following the program: 1 min 10% B; 16 min linear gradient up to 40% B; 3 min linear gradient down to 10% B. *R*-sulforaphane was detected by absorbance monitoring at 240 nm. 

### 3.9. IR of Sulforaphane

Infrared spectra were recorded on an Attenuated Total Reflectance Thermo-Nicolet AVATAR 320 AEK0200713 instrument (Perkin Elmer Instruments, Courtaboeuf, France).

### 3.10. GC/MS Analysis of Sulforaphane

GC-MS analyses of sulforaphane were carried out using a Bruker Scion SQ Premium (Bruker Daltonics, Macerata, Italy) equipped with a 30 m × 0.25 mm capillary column HP-5ms. The flow rate of the carrier gas (He) was 1 mL min^−1^. Temperature programming was from 60 °C (hold 4 min) to 200 °C at 10 °C min^−1^ (hold 1 min). The temperature of the injector and of the detector was 180 °C and 280 °C, respectively. All MS analyses were made in the electron impact (EI+) mode at 70 eV, the mass range was from 40 to 650 *m/z* and the chromatogram acquired in total ion current (TIC). 

### 3.11. Optical Rotation of Sulforaphane

Sulforaphane (13.0 mg) was weighed in a 1 mL volumetric flask and dissolved in chloroform (concentration (*c*) expressed in g/100 mL). The solution was transferred into a 1 mL cell (path length 1 dm) and the optical rotation [α]_D_ was measured at 25 °C on a Perkin-Elmer 141 polarimeter (Perkin Elmer Instruments, Courtaboeuf, France). 

## 4. Conclusions

Natural sulforaphane exists as a single *R_S_*-configurated enantiomer and this enantiopurity appears critical for its biological activity. However, only few studies up to now have evaluated the influence of the sulfoxide chirality on the chemopreventive and anticancer activity of sulforaphane. We have disclosed here an efficient and simple method for producing multi-gram quantities of this natural ITC to be used in future investigations in animal and clinical studies. 
